# Expression of Concern: LncRNA FBXL19-AS1 promotes breast cancer cells proliferation and invasion via acting as a molecular sponge to miR-718

**DOI:** 10.1042/BSR-2018-2018_EOC

**Published:** 2022-11-18

**Keywords:** breast cancer, lncRNA FBXL19-AS1, miR-718

The Editorial Office has been made aware of potential issues surrounding the scientific validity of this paper, hence has issued an Expression of Concern to notify readers whilst the Editorial Office investigates. It has been noted that this paper shares similarities by a manuscript by Jiang et al. 2018 (https://doi.org/10.1186/s13046-022-02418-x), with no shared authors. Specifically, similarities have been identified between the panels of Figure 4F (the Ki-67/sh-NC panel having been vertically flipped) and those of Jiang et al. Figure 3C (Ki67 sh-Vector and sh-TPT1-AS1 panels), and between the western blots of Figure 2F and Jiang et al. Figure 5H.

